# Bacterial IgA protease-mediated degradation of agIgA1 and agIgA1 immune complexes as a potential therapy for IgA Nephropathy

**DOI:** 10.1038/srep30964

**Published:** 2016-08-03

**Authors:** Li Wang, Xueying Li, Hongchun Shen, Nan Mao, Honglian Wang, Luke Cui, Yuan Cheng, Junming Fan

**Affiliations:** 1 Laboratory of Organ Fibrosis Prophylaxis and Treatment by Combine Traditional Chinese and Western Medicine, Research Center of Combine Traditional Chinese and Western Medicine, Affiliated Traditional Medicine Hospital of Southwest Medical University, Luzhou, Sichuan, 646000, China; 2 State Key Laboratory of Biotherapy, West China Hospital, Sichuan University, Chengdu, Sichuan, 610041, China; 3 Department of Nephrology, The Affiliated Hospital of Southwest Medical University, Luzhou, Sichuan, 646000, China; 4 College of Integrated Chinese and Western Medicine, Southwest Medical University, Luzhou, Sichuan, 646000, China; 5 Department of Nephrology, The First Affiliated Hospital of Chengdu Medical College, Chengdu, Sichuan, 610041, China; 6 Department of Nephrology, The Third People’s Hospital of Chengdu, Chengdu, Sichuan, 610041, China; 7 Department of Nephrology, Shenzhen Second People’s Hospital, The First Affiliated Hospital of Shenzhen University, Shenzhen, 518000, China; 8 Department of Nephrology, The Affiliated Traditional Chinese Medicine Hospital of Southwest Medical University, Luzhou, Sichuan, 646000, China

## Abstract

Mesangial deposition of aberrantly glycosylated IgA1 (agIgA1) and its immune complexes is a key pathogenic mechanism of IgA nephropathy (IgAN). However, treatment of IgAN remains ineffective. We report here that bacteria-derived IgA proteases are capable of degrading these pathogenic agIgA1 and derived immune complexes *in vitro* and *in vivo*. By screening 14 different bacterial strains (6 species), we found that 4 bacterial IgA proteases from *H. influenzae, N. gonorrhoeae and N. meningitidis* exhibited high cleaving activities on serum agIgA1 and artificial galactose-depleted IgA1 *in vitro* and the deposited agIgA1-containing immune complexes in the mesangium of renal biopsy from IgAN patients and in a passive mouse model of IgAN *in vitro*. In the modified mouse model of passive IgAN with abundant *in situ* mesangial deposition of the agIgA-IgG immune complexes, a single intravenous delivery of IgA protease from *H. influenzae* was able to effectively degrade the deposited agIgA-IgG immune complexes within the glomerulus, demonstrating a therapeutic potential for IgAN. In conclusion, the bacteria-derived IgA proteases are biologically active enzymes capable of cleaving the circulating agIgA and the deposited agIgA-IgG immune complexes within the kidney of IgAN. Thus, the use of such IgA proteases may represent a novel therapy for IgAN.

IgAN is the most common glomerulonehritis characterized by mesangial deposition of the IgA1-containing immune complexes^1^. In the physiological conditions, human IgA1 exhibits the O-glycosylation on the serine (Ser) and threonine (Thr) residues located in the hinge region of the IgA1 heavy chain. These O-glycan side chains exist as disaccharide with a primary N-acetylgalactosamine (GalNAc) and a secondary β1,3-linked galactose, both of which can be sialylated^2^. However, in patients with IgAN, the serum IgA1 and the deposited IgA1 within the mesangial areas are aberrantly glycosylated or galactose-deficient in its hinge region, termed as aberrantly glycosylated IgA1(agIgA1)^1^,^3^. Consistently, significant higher levels of serum agIgA1 are found in patients with IgAN^3^,^4^. The abnormally-produced agIgA1 can act as an autoimmune antigen that can be recognized by particular IgG or IgA antibodies to form the immune complexes that are prone to deposit in the mesangial areas and results in glomerular inflammation, mesanigal hypercellularity, and expansion of mesangial matrix^5^,^6^,^7^. Therefore, IgAN is considered to be an autoimmune disorder with features of progressive chronic kidney disease. However, effective treatment for IgAN remains lacking.

Increasing evidence shows that direct removal of agIgA1and its immune complexs may be a promising therapy for IgAN. It has been reported that the mesangial deposition of IgA immune complexes in patients with IgAN can be removed after the kidney is transplanted into a non-IgAN individual^8^. In a patient with severe HSPN (Henoch-Schonlein purpura nephritis), an autoimmune disorder that mimics IgAN, renal injury unexpectedly relieved after acquiring a IgA-deficiency secondary disease^6^. This observation suggests that the removal of IgA-containing immune complexes from the kidney may represent a novel therapy for IgAN. A pioneer work by Lamm and colleagues has shown that the bacteria-derived IgA protease can efficiently degrade the deposited IgA1-IgG immune complexes in a passive mouse model of IgAN^9^. However, it remains unclear whether the use of IgA proteases can effectively degrade the pathogenic agIgA1 and its immune complexes in the mesangium in patients or animal model with IgAN.

In the present study, by screening 14 bacterial strains (6 species), we first identified and characterized 4 bacterial-derived IgA proteases that were capable of decomposing the agIgA1 and agIgA1-containing immune complexes from serum and locally in the diseased kidney in patients and mouse model of IgAN. Then, we examined the therapeutic potential of a bacterial IgA protease from *H. influenzae* in a passive mouse model of IgAN.

## Results

### Identification and characterization of bacterial IgA proteases

**Figure 1:**
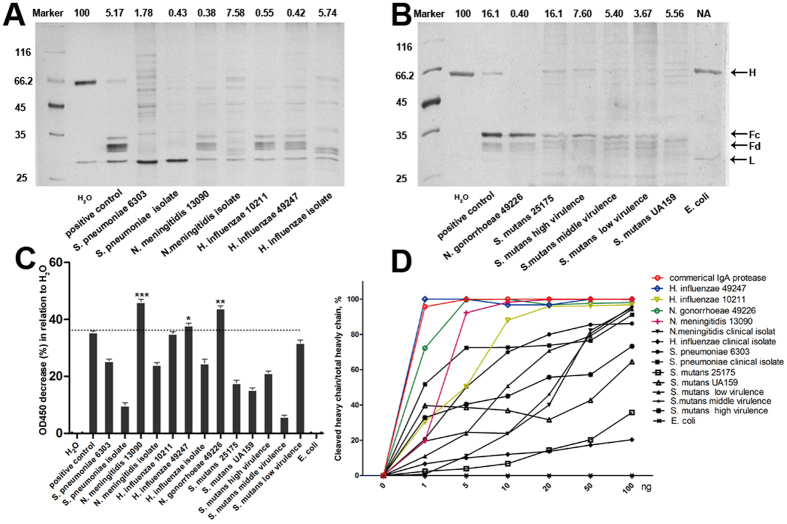
Catalytic activities of IgA proteases from different bacteria. (**A**,**B**) 0.5 μg of human IgA1 was subjected to overnight digestion by 0.05 μg of IgA proteases from indicated bacterial strains. The digestion pattern was resolved by SDS-PAGE electrophoresis. The percentage of the residual heavy chain was indicated up the lane. A commercial IgA protease derived from *N. gonorrhoeae* was used as positive control. H stands for the heavy chain of IgA1 and L the light chain. Fc and Fd are the final products of digested IgA1 heavy chain. (**C)** Quantification of catalytic activities of different bacterial IgA protease by ELISA-based assay. Data represent 4 independent replicates and each bar represents mean ± SEM for the percentage of reduction of OD value compared with the negative control (H_2_O). *P < 0.05 vs positive control. **P < 0.01 vs positive control. ***P < 0.001 vs positive control. (**D**) Dosage-dependent digestion of IgA1 by IgA proteases. 0.5 μg of IgA1 was incubated with different amount of IgA protease at 37 °C for 2 hours. The digestion mixture was subjected to SDS-PAGE analysis. The percentage of cleaved heavy chain (in relative to the negative control) was ploted. Note the ploted lines for commercial IgA protease, and proteases from *N. gonorrhoeae 49226*, *N. meningitidis 13090*, *H. influenzae 49247* and *10211* were highlighted colorfully.

In order to obtain the bacterial IgA proteases with higher catalytic activities, we screened 14 bacterial strains from 6 species which were reported to show higher IgA protease activity or can be easily acquired^10^. Crude IgA proteases were purified from each bacterial culture supernatant with a procedure consist of ultrafiltration, dialysis, hydrophobic interaction and ion exchange chromatography as described in methods. Human myeloma IgA1 was exposed to IgA proteases from 14 bacterial stains overnight at 37 °C, respectively. SDS-PAGE analysis of the digestion product detected that, compared with the control (H_2_O), more than 90% of the heavy chain of IgA1 can be cleaved into Fc and Fd fragment by almost all IgA proteases employed except the one from *E. coli* in which no enzymatic activity was observed (Fig. 1A,B). While most of the bacteria-derived IgA proteases showed variable IgA1 degradation activities, those from *H. influenzae, N. gonorrhoeae, N. meningitidis* and *S. pneumoniae* showed relative higher activity. Furthermore, we also found that bacteria from the same species but different strains also exhibited distinct activity. For example, the IgA protease activity of commercially available *N. meningitidis* (13090) and *H. influenzae* (10211 and 49247) was more robust than those from clinical isolates. However, IgA proteases from *S. mutans* showed overall weaker catalytic abilities.

To further confirm the above observations, an ELISA-based method was used to quantitatively determine the IgA protease activities by measuring the residual IgA1 after 2 hours’ IgA protease digestion in the reaction mixture. As shown in Fig. 1(C), among the IgA proteases employed, proteases from the *E. coli* or *S. mutans* strains showed no or relative low IgA1 degradation activities, respectively. IgA proteases from *N. gonorrhoeae 49226, H. influenzae 10211* and *49247*, and *N. meningitidis* 13090 exhibited the greatest IgA1 cleavage capacities. Thus, the quantitative ELISA data coincided well with SDS-PAGE results.

We further examined the dosage-dependent activity of IgA proteases from different bacteria on IgA1 in a shorter time (2 hours) and found that IgA proteases from *N. gonorrhoeae 49226*, *N. meningitidis 13090*, *H. influenzae 10211* and *49247* reached almost 100% degradation of the IgA1 at smaller dose. The left IgA proteases showed gradually increased cleavage product when a larger dose of enzyme was provided except that from *E. coli* (Fig. 1D). Taken together, IgA proteases from *N. meningitidis 13090*, *N.gonorrhoeae 49226*, *H. influenzae 10211* and *49247* were selected for downstream analysis given their relatively higher activity.

To achieve high purity of IgA protease for deep investigation, the crude IgA proteases of *N. meningitidis 13090*, *N. gonorrhoeae 49226*, *H. influenzae 10211* and *49247* were further fractioned by gel filtration to remove impurity. The final purity was confirmed by SDS-PAGE analysis (Supplementary Figure S1). IgA proteases from *H. influenzae 10211* and *49247* have a molecular weight slight larger than 70 kDa. IgA protease from *N. meningitidis 13090* is about 100 kDa. *N.gonorrhoeae 49226* demonstrated a IgA protease with comparable size with commercial IgA protease derived from the same species. The specificity of these proteases were confirmed by overnight co-incubation with human IgG or bovine serum albumin (BSA) without trace degradation (Supplementary Figure S2), excluding contamination of additional non-specific protease. Coincide with the published data, all the four IgA proteases showed sensitivity to PMSF and demonstrated DTT-independent activity, suggesting their serine-type protease identity (Supplementary Figure S3)^10^.

### *In vitro* ability of bacterial IgA proteases to degrade the galactose-deficient IgA1 and agIgA1 from patients with IgAN

**Figure 2:**
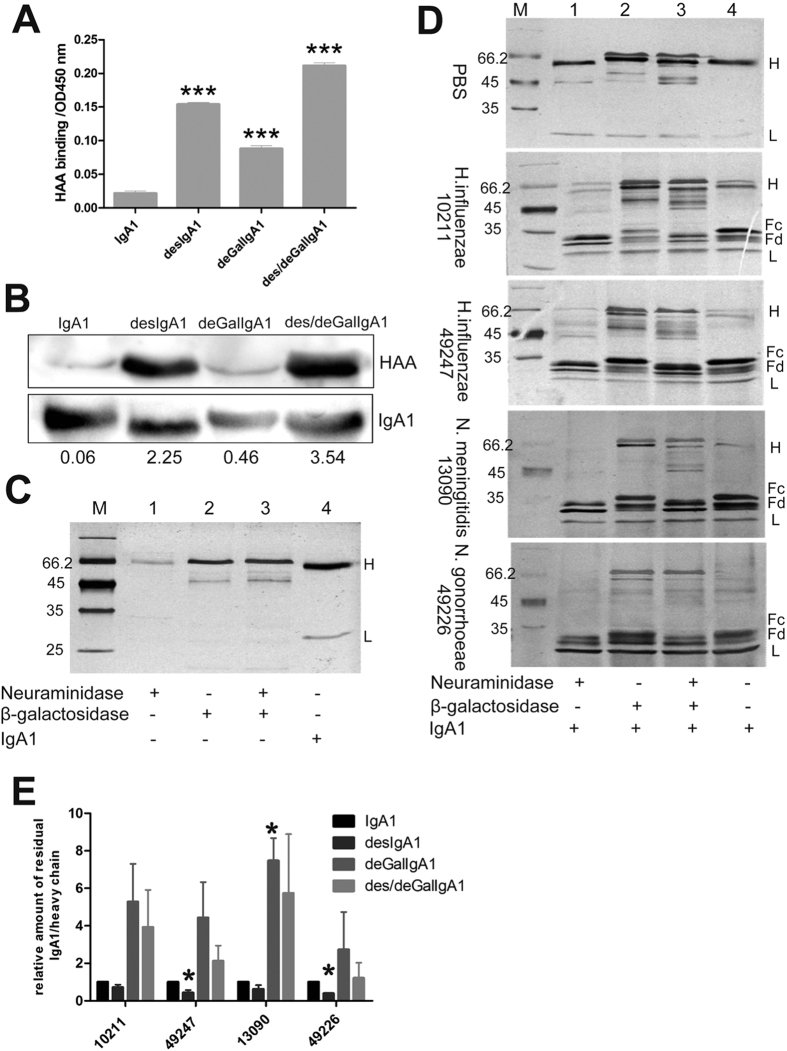
*In vitro* degradation ability of IgA proteases on deglycosylated IgA1. (**A)** Determination of glycosylation level of IgA1 after treatment with different deglycosylation enzyme (β-galactosidase and neuraminidase) by HHA-based ELISA. Each sample was assayed in triplicate and data was presented as mean ± SEM. ***P < 0.001 versus IgA1 group. (**B**) Western blots for determination of glycosylation level of modified IgA1 using biotin-labeled HAA to recognize GalNAc-exposed IgA1 (upper panel) and IgA1 specific antibody to detect total IgA1 (lower panel). Number below the lanes indicated the relative abundance of Galactose-deficient IgA1. The full-length blots were presented in Supplementary information (Figure S5). (**C)** SDS-PAGE analysis of neuraminidase, β-galactosidase and IgA1 without treatment with the IgA protease. (**D**) SDS-PAGE analysis of IgA1 pre-treated with different deglycosylation enzymes and digested with IgA proteases from *H. influenzae* 10211 and 49247, *N. meningitidis* 13090 and *N. gonorrhoeae* 49226. PBS was used as a control. DesIgA1, desialylated IgA1. DeGalIgA1, degalactosylated IgA1. (**E**)Quantitative analysis of the residual IgA1 (heavy chain) in Panel **D**. Data represent for 3 independent experiments. *P < 0.05 vs IgA1 group.

To determine whether purified bacterial IgA proteases can decompose galactose-deficient IgA1, we firstly deglycosylated the human IgA1 with neuraminidase or β-galactosidase or both to generate artificially desialylated IgA1 (desIgA1), degalactosylated IgA1 (deGalIgA1) and IgA1 of desialylation and degalactosylation (des/deGalIgA1), respectively. The glycosylation status was further confirmed by *Helix aspersa* agglutinin (HAA)-based ELISA and western blot analysis, which can specifically recognize the exposed GalNAc (Fig. 2A,B)^11^. Removal of either galactose or terminal sialic acid of IgA1 vigorously increased HAA binding capacity, which was further enhanced by combined treatment of IgA1 with neuraminidase and β-galactosidase (Fig. 2C).

Next, the deglycosylated IgA1 derivatives were employed to assay IgA protease activity of the above four selected bacteria. Treatment of the 4 IgA1 variants with any of the four IgA proteases showed significant cleavage of the heavy chain. However, PBS had no effect on the integrity of heavy chain in the same reaction time (Fig. 2D). Interestingly, we found that, compared with the normal IgA1 substrate, desialylation increased the sensitivity of IgA1 to IgA proteases, especially those from *H. influenzae 49247* and *N. gonorrhoeae 49226* (Fig. 2D lane 1, 4 and Fig. 2E). However, degalactosylation (deGalIgA1) notably decreased the degradation abilities of all 4 IgA proteases at different extent. And the decreased activity can be slightly reversed by combined deglycosylation treatment (des/deGalIgA1) (Fig. 2E). This suggested that deficiency of galactose but not sialic acid impaired sensitivity of IgA1 to IgA protease. Nevertheless, the majority of the deGalIgA1and des/deGalIgA1 substrate can still be decomposed by all 4 IgA proteases. These findings indicated that although galactose-deficiency can partially inhibit the enzymatic activity, the selected bacterial IgA proteases were capable of degrading galactose-deficient IgA1 substrate.

**Figure 3:**
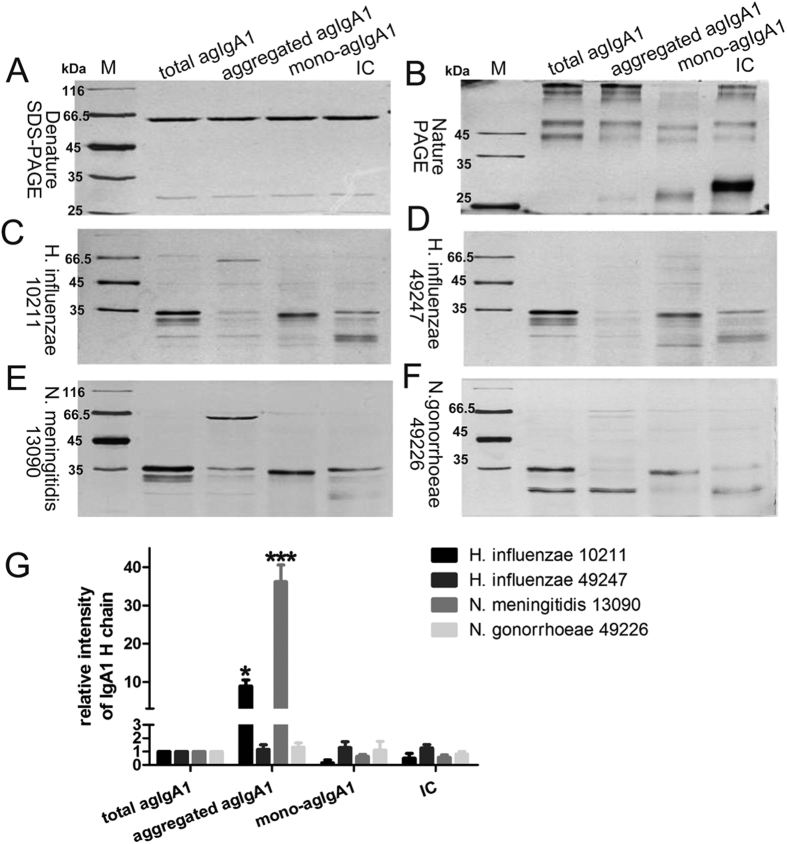
*In vitro* decomposition of agIgA1 from IgAN patient serum by bacterial IgA proteases. agIgAs of different conformation were resolved in denature (**A**) and nature (**B**) PAGE gel. agIgAs of variable conformation were treated with IgA proteases from *H. influenzae* 10211 (**C**) and 49247 (**D**), *N. meningitidis* 13090 (**E**) and *N. gonorrhoeae* 49226 (**F**) followed by SDS-PAGE analysis. Total agIgA1 stands for total serum agIgA1and IC for agIgA1-IgG immune complexs. (**G**) Quantitative analysis of the residual IgA1 (heavy chain) in (**C–F**). Data represent for 3 independent experiments. *P < 0.05 vs total agIgA1 group, ***P < 0.001 vs total agIgA1 group.

Because IgA1 exists in forms of monomer, dimer, and oligomer (or polymer) in the physiological conditions and agIgA1 also exists as circulating IgA1-IgG immune complexes in patients with IgAN, we next determined the catalytic activities of the selected 4 bacterial IgA proteases on agIgA1 of different conformation. agIgA1 was purified from the sera of IgAN patients whose aberrant glycosylation status were validated by HAA binding-based ELISA assay and western blot (Supplementary Figure S6). The purified agIgA1 was then subjected to a size-restricted chromatography to separate the monomeric agIgA1 (mono-agIgA1) from the polymeric IgA1 (pIgA1) (Supplementary Figure S6). Since the recovery of pIgA1 is very low, the pIgA1 was mimicked by heat-mediated aggregation of mIgA1 (aggregated IgA1) which demonstrated similar pathogenic effect to pIgA1, like increased binding ability to mesangial cell and stimulation of proliferation^12^. We prepared the IgA1-IgG immune complexes (IC) by incubating the purified agIgA1 with anti-human IgA antibody. The IgA1 and IgG components of the immune complexs were confirmed by western blot using species and isotype-specific IgA1 and IgG antibody (Supplementary Figure S4). As shown in Fig. 3(A,B), denatured SDS-PAGE and natural PAGE electrophoresis analysis revealed that the mono-agIgA1 formed distinct conformations compared with total agIgA1, aggregated agIgA1 and IC, and could be non-differentially degraded by the 4 bacterial IgA proteases (Fig. 3C–G). Still, no different degradation ability was observed on total agIgA1 and IC by all 4 proteases. However, although IgA proteases from *H. influenzae* 49247 and *N. gonorrhoeae* 49226 were able to efficiently digest aggregated agIgA1 (Fig. 3C–F), IgA proteases from *H. influenzae 10211* and *N. meningitidis 13090* shown impaired activities on aggregated agIgA1 (Fig. 3C,E,G).

**Figure 4:**
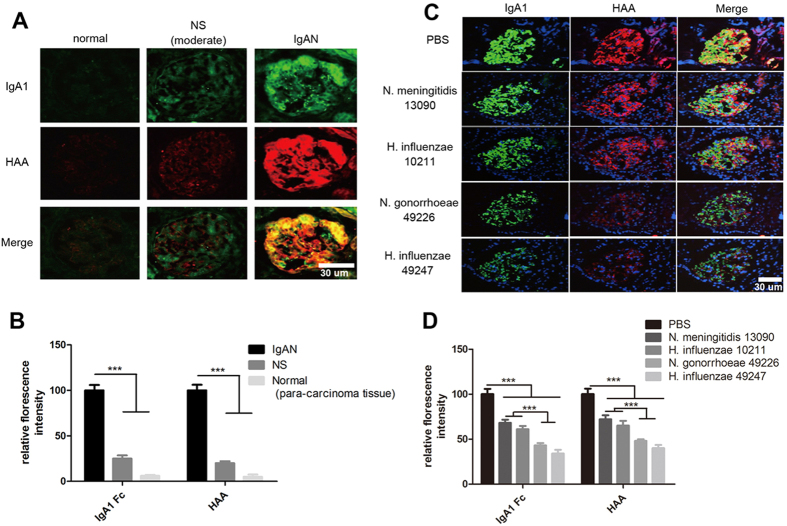
*In vitro* decomposition of agIgA1 deposited in glomeruli of patient with IgAN by IgA proteases. (**A)** Glomerular agIgA1 deposition in patients with IgAN was detected by immunofluorescence. Normal kidney tissues were obtained from para-carcinoma tissue and non-IgAN associated renal biopsy samples from patients with moderate nephritic syndrome (**NS, moderate**). (**B**) Quantitative analysis of the glomerular immunofluorescent intensity of IgA1 and HAA in panel **A**. The relative fluorescence intensity was normalized to IgAN group with assigned intensity value of 100. ***P < 0.001. (**C**) Consecutive renal biopsy sections from a patient with IgAN were treated with indicated IgA protease or PBS at 37 °C for 4 hours, respectively, following by fluorescent IgA1 staining and HAA binding assay. (**D**) Quantitative analysis of the relative immunofluorescence in panel **C**. The relative fluorescence intensity was normalized to PBS group with assigned intensity value of 100. ***P < 0.001. Each image represents for 5 independent biopsies in (**A**,**C**). And each bar represents data from at least 10 glomeruli of 5 biopsy sample in (**B**,**D**).

We next determined whether bacterial IgA proteases can degrade the deposited IgA1 in the glomerular mesangium of IgAN. Compared with kidney tissue sections from patients with nephritic syndrome and normal kidney tissue (para-carcinoma tissue of kidney cancer), a massive agIgA1 deposition in glomeruli of patient kidney biopsy with IgAN was detected by immunofluorescence with anti-human IgA1 antibody and HAA binding (Fig. 4A,B). Incubation of the consecutive kidney biopsy sections of IgAN with the IgA proteases from *H. influenzae* 49247 and *N. gonorrhoeae* 49226 resulted in effective decomposition of the deposited IgA1 in glomeruli (Fig. 4C,D). Compared with the control group, treatment with the *N. gonorrhoeae* 49226-derived IgA protease resulted in a decrease of IgA1 staining and HAA binding intensity to 43% and 48%, which was further enhanced by treatment with the IgA protease from *H. influenzae* 49247 to 34% and 40% (Fig. 4C,D). Whereas, IgA proteases from *H. influenzae 10211 and N. meningitidis* 13090 showed significantly lower degradation capacity (Fig. 4C,D).

### *In vitro* and *in vivo* degradation of IgA1 immune complexes in a passive mouse model of IgAN

**Figure 5:**
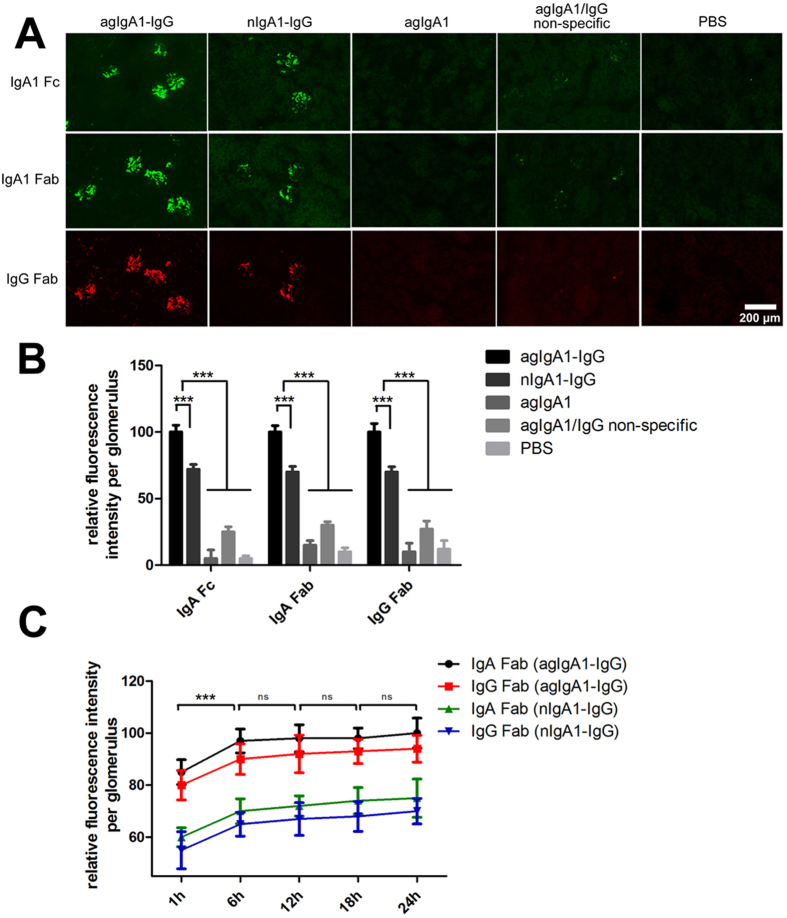
Characterization of the passive IgAN mouse model. (**A)** Deposition of IgA-associated immune complexes within mouse glomeruli after 6 hours post-injection of immune complexs (agIgA1-IgG, nIgA1-IgG) or agIgA1 or mixture of free agIgA1 and non-specfic IgG (agIgA1/IgG non-specific). The deposited IgA1 and IgG in glomeruli were detected by anti-human IgA1 (Fc and Fab fragment) or anti-goat IgG (Fab fragment) immunofluorescence. Image represents for sections from 10 mice in each group. (**B**) Quantitative analysis of the relative immunofluorescence in panel **A**. Relative fluorescence intensity was normalized to agIgA1-IgG group with assigned value of 100. (**C**) Quantitative analysis of immunofluorescent intensity of IgA1 and IgG components in passive IgAN mouse model at indicated time point post-injection of immune complexs. The relative fluorescence intensity was normalized to the glomerulus showing greatest fluorescence with assigned intensity value of 100. Data represents 60 glomeruli on 20 sections from 10 mice for each group in (**B**,**C**). ***P < 0.001. ns, no significance. agIgA1, aberrantly glycosylated IgA1; nIgA1, normal IgA1.

We next studied the functional role of bacterial IgA proteases in a pathological condition with a passive mouse model of IgAN in which a massive glomerular deposition of IgA1-IgG immune complexes has been described by Lamm^9^. As shown in Fig. 5(A,B), a single dose of intravenous injection of IgA1-IgG immune complexs (700 μg per mouse, molar ratio 1:1) containing IgA1 derived from normal individual (nIgA1-IgG) or IgAN patient serum (agIgA1-IgG) resulted in a massive deposition of the immune complexes in glomeruli at 6-hour post-injection. However, animals injected with agIgA1 alone, control saline and the mixture of free agIgA1 and non-specific IgG (agIgA1/IgG non-specific) developed no or minimal immune complexes deposition in glomeruli (Fig. 5A,B). Notably, mice received intravenous injection of agIgA1-IgG immune complexes developed more intensive immune complexs deposition than those received nIgA1-IgG. agIgA1-IgG increased the amount of deposited immune complexs by nearly 20% than nIgA1-IgG, suggesting that the agIgA1-containing immune complexes are more prone to deposit in the mesangium. On the other hand, we observed that accumulation of immune complexs in glomeruli took place rapidly in the first 6 hours post-injection and increased somehow slowly beyond time point of 6-hour (the increase was slight and not statistically significant between neighboring check point). This suggested that the majority of the injected immune complexs deposited in the glomeruli in the first 6 hours (Fig. 5C). Although there was no significant glomerular hypercellularity and inflammatory cell accumulation, this acute and short-termed model can well mimic the deposition of human-derived IgA1 containing immune complexs in glomerulus.

**Figure 6:**
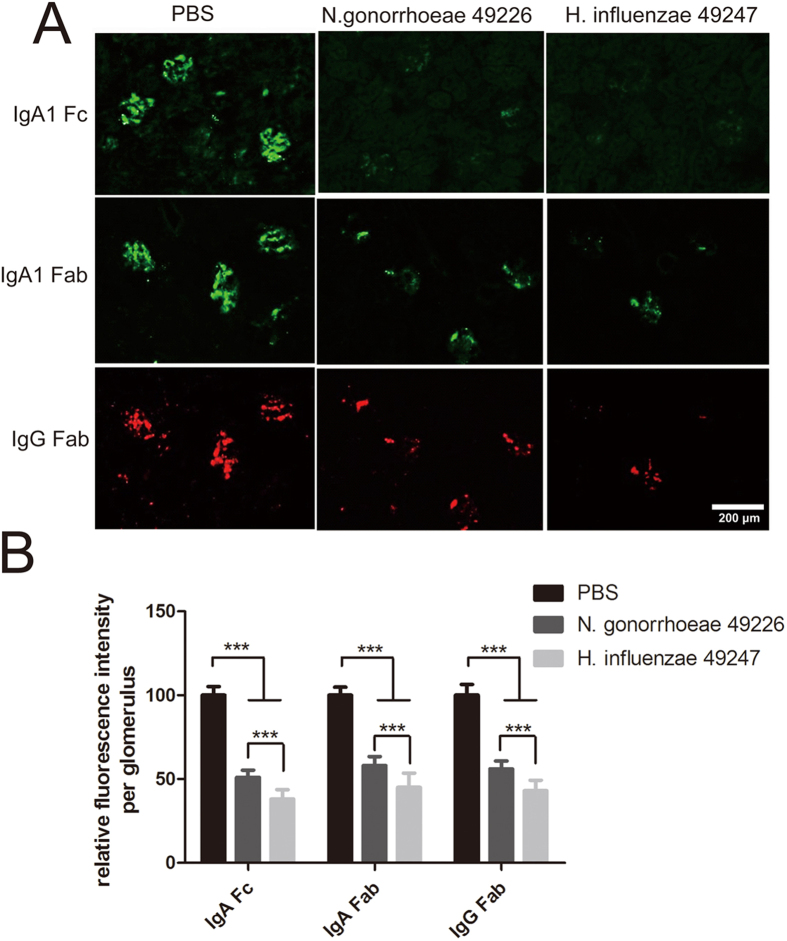
*In vitro* degradation of agIgA1-IgG immune complexs in passive IgAN kidney by IgA proteases. (**A**) Kidney sections from IgAN mouse model induced by agIgA1-IgG immune complexs were treated with IgA proteases from *H. influenzae* 49247 or *N. gonorrhoeae* 49226 or PBS at 37 °C for 4 hours. The digested tissues were immunostained against IgA1 Fc and Fab fragment (green) and IgG Fab fragment (red). Image represents 20 sections each group. (**B**) Quantitative analysis of fluorescence intensity of IgA1 and IgG components deposited in glomeruli from passive IgAN mouse model. Data was represented at least 60 glomeruli of 20 section each group. ***P < 0.001.

To investigate the capacity of bacterial IgA proteases to degrade the agIgA1 immune complexs that deposited in the kidney with passive IgAN, kidney sections from IgAN mouse induced with agIgA1-IgG immune complexs were treated with the IgA protease from *H. influenzae* 49247 or *N. gonorrhoeae* 49226. As illustrated in Fig. 6, incubation with both two bacterial IgA proteases for 4 hours caused a remarkable degradation (about 50%) of deposited IgA1 in the kidney sections compared with the control group treated with PBS for the same time. Moreover, the IgG component (Fab fragment) of the immune complexs was also removed by both bacterial IgA proteases. However, IgA protease from *H. influenzae* 49247 showed a more robust activity than that of *N. gonorrhoeae* 49226 for the removal of deposited immune complexs from the glomerulus (60% vs 40%) of the diseased kidney *in vitro* (Fig. 6A). Therefore, IgA protease from *H. influenzae* 49247 was used for downstream *in vivo* study.

**Figure 7:**
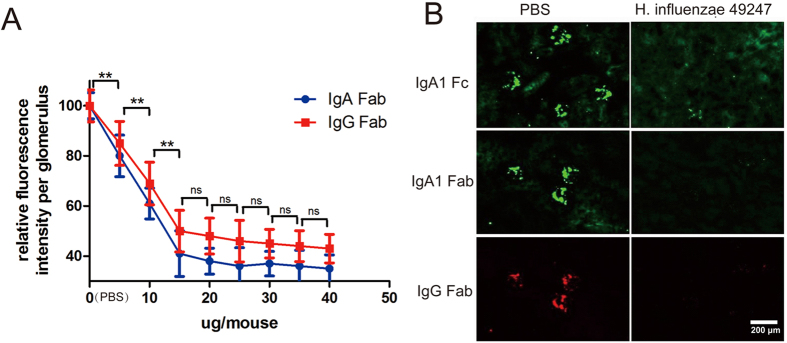
*In vivo* Clearance of deposited agIgA-IgG immune complexs by bacterial IgA protease of *H. influenzae* 49247 in passive IgAN mouse model. (**A**) Passive IgAN mice were intravenously delivered indicated amount of IgA protease from *H. influenzae* 49247 or PBS 6-hour post model construction. The mice (n = 5 for each group) were sacrificed 2 hours later and relative fluorescent intensity of residual immune complexs was plotted. Data represents 20 glomeruli on 10 sections of 5 mice each group. **P < 0.01 for both IgA Fab and IgG Fab. ns, no significance for both IgA Fab and IgG Fab. (**B**) Representative images of immunofluorescence of human IgA1 Fc, Fab (green) and goat IgG Fab (red) fragment in passive IgAN mice treated with 15 μg of *H. influenzae* 49247 IgA protease.

We next examined the therapeutic potential of the purified bacterial IgA protease from *H. influnzae* 49247 in the IgAN mouse model which was induced by intravenously administration of the agIgA1-IgG immune complexs as described above. 6 hours after the immune complexs injection, mice were further intravenously delivered various dosages of the IgA protease from *H. influenzae* 49247. 2 hours later, mice were sacrificed and frozen kidney sections were examined for immune complexs by immunofluorescence. As shown in Fig. 7(A), compared with the PBS group, injection of the IgA protease significantly reduced the amount of deposited IgA-IgG immune complexs |in a dosage-dependent manner as indicated by immunostaining of IgA (Fc and Fab) and IgG (Fab) fragment, respectively. And the most steep immune complexs clearance occurred in a dosage less than 15 μg (per mouse). Treatment with 15 μg of *H. influenzae* 49247 IgA protease reduced the calculated deposition of IgA1 component by 62% for Fc fragment (*p* < 0.001 versus saline group) and 59% for F(ab’)_2_ fragment (*p* < 0.001 versus saline group), respectively. Meanwhile, deposition of IgG Fab fragment also decreased by 50% (*p* < 0.001 versus saline group), suggesting that both IgA and IgG components were gradually removed from the glomeruli by the IgA protease (Fig. 7A,B).

Furthermore, to explore the possible toxicity of IgA protease, larger dose of *H. influenzae* 49247 IgA protease (both 30 and 60 μg per mouse respectively) were intravenously delivered and no significant histological abnormality of major organs, including liver, kidney, spleen and intestine, was observed 24 hours post injection (Supplementary Figure S7). In line with the histopathology data, these mice showed normal serum level of albumin, glutamic-oxaloacetic transaminase, glutamic-pyruvic transaminase, blood urea nitrogen, creatinine, cystatin C and glomerular filtration rate compared with the control mice treat with PBS, implying normal liver and kidney function. Meanwhile, no difference of serum triglyceride and amylase level were found, indicating normal lipid metabolism and absence of acute pancreatitis (Supplementary Table S1). Thus, the injection of IgA protease showed no obvious toxicity in mice, at least at the dosage tested in our experiment.

## Discussion

Given the important role of agIgA1 in the pathogenesis of IgAN, the present study provided clear evidence that targeting for degradation of both circulating agIgA1 and deposited agIgA-associated immune complexes within the diseased kidney with bacteria-derived IgA proteases may represent a novel and potential therapy for IgAN.

On the basis of the pioneer work by Lamm’s group, the most significant finding in this study was the identification of 4 bacteria-derived IgA proteases from 14 different bacterial strains (6 species) that were capable of cleaving the artificial galactose-depleted IgA1, pathogenic agIgA1 in distinct conformations, and the deposited agIgA1-containing immune complexes locally in the glomerulus in patients with IgAN and in the passive mouse model of IgAN. Of them, IgA proteases from *H. influenzae* 49247 and *N. gonorrhoeae* 49226 produced better enzymatic activities than those from *H. influenzae* 10211 and *N. meningitidis* 13090 in term of the cleavage of artificial galactose-depleted IgA1, different forms of agIgA1 and its immune complexs. However, *H. influenzae* 49247-derived IgA protease performed better than that from *N. gonorrhoeae* 49226 in term of the degradation of deposited agIgA1 immune complexs, which was also selected for *in vivo* therapeutic analysis in Lamm’s work^9^.

In Lamm’s study, bacterial IgA protease was proved to remove IgA1 immune complexs *in vitro* and *in vivo*^9^. However, since the aberrant glycosylation or galactose-deficiency in the hinge region of IgA1 molecule may influence its sensitivity to IgA protease-mediated cleavage, it is a concern on the ability of bacterial IgA proteases to cause the degradation of the pathogenic IgA1 such as agIgA1 and agIgA1-containing immune complexs. Indeed, the enzymatic hydrolytic site by IgA protease is located at the same region of IgA1 molecule subjected to glycosylation modification. Glycosylation modification usually confers the glycoprotein improved resistance to proteases and thus enhances its stability^13^. It is reported that depletion of terminal sialic acid increases the sensitivity of IgA1 to IgA protease, which keeps in line with our data (Fig. 2)^14^. However, it’s controversial on the impact of absence of intact disaccharide chain on the susceptibility of IgA1 to IgA protease^14^,^15^. Thus, the glycosylation status of IgA1 hinge region has complicated influence on the sensitivity to IgA protease. Results from the present study provided the evidence that galactose-depletion of IgA1 indeed impaired the cleavage activity at a IgA protease-dependent manner. Nevertheless, the majority of the artificial galactose-deficient IgA1 can still be effectively degraded by IgA proteases as seen in the present study.

More significantly, we also found that the administration of an IgA protease from *H. influenzae* was capable of removing the deposited agIgA1-IgG immune complexes from the glomerulus in the modified passive mouse model of IgAN *in vivo*. This finding provided a direct evidence for bacteria-derived IgA protease as a specific and effective therapeutic agent for IgAN clinically. In this study, we used the pathogenic agIgA1-IgG immune complexes from patients with IgAN to induce a passive model of IgAN and examined the degradation activity of IgA protease on the deposited agIgA1-containing immune complexes *in vivo*. We found that the use of agIgA1 immune complexes resulted in a greater deposition of immune complexs in glomeruli than the normal IgA1-derived immune complexes used in Lamm’s study, suggesting the agIgA1-IgG immune complexes exhibit greater affinity to mesangial cells and agIgA1-IgG immune complexs-induced mouse could be a better passive mouse model to mimic the immune complexs deposition in a pathologically similar condition. This is consistent with the notion that heat-aggregated IgA1 from IgAN patient has higher binding capacity and stronger biological effects than that from normal individuals^12^. The ability of single injection of *H. influenzae-*derived IgA proteases to effectively remove the pathogenic IgA1 and agIgA1-IgG immune complexes from the diseased kidney provided a clear evidence for the therapeutic potential of IgA protease for IgAN. Furthermore, no significant toxicity of IgA protease was observed in this study. Thus, results from the present study provided new evidence supporting the therapeutic potential for IgAN by using IgA proteases.

It should be pointed out that, as the short-termed passive IgAN model showed no obvious kidney function impairment and other animal models ever reported are not based on a pathogenicity of human IgA1-containing immune complexs deposition, renal protection role of injected IgA protease, like decreased blood creatinine and urea nitrogen, can’t be directly observed in current passive IgAN model. A larger dosage of immune complexs injection or repeated injection can be tried to copy the kidney injury of IgAN in future research. It was reported that in rat glomerulonephritis model, non-specific protease mixture injection relieved disease symptom without significant toxicity^16^,^17^. We also provided the data to validate the safety of IgA protease, a more specific protease, in mouse at least for the dosage tested in our study. However, since lower-ordered animals contain no IgA1 variant sensitive to IgA protease, the side effects of injected IgA protease should be further assayed in IgA1-containing animal, like monkey. Furthermore, as IgA protease can not only degrade agIgA1 and its immune complexs, but also normal IgA1 which is important to mucosal immunity, risk still remains in future clinical application. To minimize the disturbance of normal IgA1-mediated immunity, the drug format can be modified as particle of nanometer level to target the kidney more specifically^18^,^19^. In conclusion, bacterial IgA protease presents fantastic potential for IgAN treatment while there are still a lot of problems remained to resolve.

## Methods

### Bacteria and IgA protease purification

Bacterial strain of *N. gonorrhoeae* (ATCC49226) were purchased from Fuxiang Bio-tech Ltd (Shanghai, China). *H. influenzae* with unkonwn serum type (ATCC49247), *H. influenzae* serum type b (ATCC 10211), *S. pneumoniae* serum type 3 (ATCC 6303™), *N. meningitidis* serum type B (ATCC 13090™) were bought from Chuanxiang Bio-tech Ltd (Shanghai, China). *S. mutans* serum type C (ATCC 25175™), *Streptococcus mutans* with unknown serum type (UA159), clinical isolates of *H. influenzae*, *N. meningitides*, *S. pneumoniae*, *S. mutans* (low, moderate and high virulence), *E. coli* were kindly provided by School of Stomatology of Southwest Medical University. The detailed culture conditions for each bacterial were listed in Supplementary Table S2.

For IgA proteases isolation, 1 liter of culture medium of each bacteria at exponential growth phase was cleared by centrifugation and concentrated to about 200 ml by ultrafiltration. By slowly adding equal volume of saturated (NH_4_)_2_SO_4_ solution (in 50 mM Tris-HCl, pH 7.5) with string, crude protein was precipitated overnight. The precipitation was re-dissolved in phosphate buffer (12.2 mM Na_2_HPO_4_, 7.75 mM NaH_2_PO_4_) containing 0.5 M (NH_4_)_2_SO_4_ and applied to a hydrophobic HiTrapTM phenyl sepharose FF (LS) column (GE healthcare, USA) balanced with the same buffer. After wash with 5 fold volume of balance buffer, the bond protein was eluted with the same phosphate buffer without (NH_4_)_2_SO_4._ Eluted fractions were assayed for IgA protease activity. Fractions containing IgA protease activity were pooled and dialyzed to 50 mM Tris-HCl (pH 8.5) by ultrafiltration. The resulted crude IgA protease was further absorbed to a Toyopearl SuperQ-650M (TOSOH, Japan) anion exchange chromatography column (16 mm by 300 mm) balanced with 50 mM Tris-HCl (pH 8.5). After wash with 10 fold volume of the balance buffer, protein was eluted with gradually increased linear gradient of 10–400 mM NaCl (in 50 mM Tris-HCl, pH 8.5). Fractions containing IgA protease activity were pooled, dialyzed to PBS and concentrated by ultrafiltration. Protein concentration was quantified with a BCA method (Beyotime Biotech, China) and adjusted to 1 mg/ml with 50 mM Tris-HCl (pH 8.5) or PBS supplemented with 50% glycerol and stored at −20 °C in aliquot. To achieve high purity of IgA protease of *H. influenzae* 49247, *H. influenzae* 10211, *N. gonorrhoeae* 49226 and *M. mengingitidis* 13090, the concentrated IgA protease solution prepared by above selection chromatography was further fractioned by a gel filtration column (molecular sieve column, 22 mm by 1000 mm) equipped with Sephacryl S-200HR resin (GE healthcare, USA). The mobile phase was PBS (plus 0.01% NaN_3_). Fractions showing IgA protease activity were pooled and concentrated by ultrafiltration. Purity of purified IgA protease was confirmed by SDS-PAGE analysis (Supplementary Figure S1). All purification steps should be performed at 4 °C as possible.

### IgA1 Purification from Human Sera

Sera from healthy individuals and patients of IgAN whose glycosylation status was confirmed by HAA binding assay and western blot were pooled and total serum protein was precipitated with (NH_4_)_2_SO_4_. After re-dissolution in 0.02 M Tris-HCl (pH 8.0) and dialysis, the resulting solution was passed through a 0.22 μm filter and subjected to DEAE52-Cellulose chromatography. Protein was eluted with gradually increased concentration of NaCl (20–200 mM in 0.02 M Tris-HCl (pH 8.0)). The eluted fractions containing IgA1 were pooled. The IgA1 was absorbed with Jacalin affinity chromatography column and eluted with 0.1 M melibiose in PBS. Again, the eluted fractions containing IgA1 were combined and concentrated by dialysis against PEG2000. To separate monomeric IgA1 from polymeric IgA1, the concentrate was applied to Sephadex G-200 size-restricted chromatography column (Supplementary Figure S6). The protein fractions corresponding to pIgA1 and mIgA1 were collected respectively and further concentrated by dialysis against PEG2000. The protein concentration was determined with BCA method. In this study, all the human materials including blood and kidney specimen (elsewhere) were obtained and used under the informed consent which was completely understood and agreed by all subjects enrolled in this project. The study involved with human tissue was approved by Ethics Committee of Southwest Medical University and all the manipulation were performed in accordance with the Helsinki Declaration.

### Aggregated agIgA1 and IgA1-IgG immune complexs

Monomeric agIgA1 was incubated in water bath of 63 °C for 2.5 hours and chilled on ice followed by centrifugation at 4 °C 12000 rpm for 5 min to remove any insoluble precipitation^12^. Sephadex G-200 chromatography analysis indicated only one protein elution peak corresponding to pIgA1, suggesting a relative homologous aggregation status. For generation of IgA1-IgG immune complexs, the purified agIgA1 (from IgAN patients) or normal IgA1 (from healthy individuals) was incubated with goat anti-human F(ab)_2_ antibody (AffiniPure F(ab’)_2_ Frag Goat Anti-Human IgG, F(ab’)_2_ Frag Specific, Jackson ImmunoResearch, USA) at a molar ratio of 1:1 at room temperature for 5 min. The immune complexs was stored at 4 °C. For preparation of “agIgA1/IgG non-specific” in Fig. 5, agIgA1 was mixed with non-specific IgG (ChromPure Goat IgG. F(ab’)_2_ Fragment, Jackson ImmunoResearch, USA) as above. The existence of IgA1 and IgG components in immune complexs was confirmed by western blot to probe the precipitation from agglutination of IgA1 and IgG (Supplementary Figure S4).

### Degalactosylation and Desialylation of IgA1

For desialylation, IgA1 was treated with neurminidase (0.1 U per mg IgA1, Sigma, USA) in buffer containing 100 mM sodium acetate plus 2 mM CaCl_2_ (pH 5.0) for 6 hours at 37 °C. For degalactosylation, IgA1 was incubated with β-galactosidase (0.1 U per mg IgA1, Sigma, USA) in buffer containing 10 mM Tris-HCl (pH 7.3), 10 mM MgCl_2_, 1 mM β-mercaptoethanol supplemented with 0.1 mg/ml BSA. The deglycosylated IgA1 was dialyzed against ddH_2_O, lyophilized and re-dissolved in ddH_2_O. For preparation of des/deGalIgA1, desialylation preceded the degalactosylation reaction.

### *In vitro* IgA protease Activity Assay

0.5 μg of human myeloma-derived IgA1 (Merck KGaA, Germany) or deglycosylated IgA1 or IgAN patient-derived IgA1 of different conformation was incubated with 0.05 μg of purified IgA protease from indicated strains in 50 mM Tris-HCl, 100 mM NaCl and 1 mM EDTA (pH 7.5) at 37 °C for indicated time followed by SDS-PAGE electrophoresis and silver staining analysis. For dosage-dependent activity analysis, 0.5 μg of human IgA1 was treated with different amount (1, 5, 10, 20, 50, 100 ng) of IgA protease at 37 °C for 2 h and subjected to SDS-PAGE analysis as above. A commercially available recombinant IgA protease derived from *N. gonorrhoeae* was used as positive control (MoBiTec, Germany) where indicated. The gray intensity of heavy chain band was quantified by Image J 1.47V software (NIH, USA) to represent the residual IgA1 amount.

### ELISA

The 96-well plate was coated with 0.25 μg of goat F(ab)’_2_ anti-human IgA Kappa (InvivoGen, Hongkong) at 4 °C overnight. After washing with PBST (PBS plus 0.1% tween 20). Plate was blocked with 1% BSA in PBST for 1 h at 37 °C. For ELISA-based IgA protease activity determination, 0.5 μg of human IgA1 was first digested with indicated IgA protease or saline for 2 h at 37 °C. After dilution of the digestion mixture, a total amount of 10 ng of equivalent original IgA1 containing diluted mixture was added to the above coated well. After incubation at 37 °C for 2 h, the plate was washed. HRP-conjugated goat anti-human IgA antibody (1:8000, SouthernBiotech, USA) was used to recognize the undigested IgA1 substrate at 37 °C for 1 h. After regular washing with PBST, chromogenesis was conducted in 100 μl of TMB solution for proper time and stopped by addition of 100 μl of 2 M H_2_SO_4._ Absorbance at 450 nm was determined with a BioRad Model 680 spectrophotometer (USA). The activity of the IgA protease was presented by the percentage of the decreased absorbance value compared to the control (saline).

For total serum IgA quantification, the serum was diluted at 1:10E6 fold and 100 μl of diluted sample was subjected to ELISA assay as above without IgA protease digestion. For HAA-based ELISA assay, the bond human serum IgA1 was treated with neuraminidase (Roche, Germany) or β-galactosidase (Sigma, USA) as previously described^20^. After washing, 100 μl of biotinylated helix aspersa agglutinin (HAA) lectin (1:250, Sigma, USA) was used to recognize the galactose-deficient IgA1 for 2 h at 37 °C followed by washing. Then 100 μl of HRP-conjugated avidin (1:10000, Sigma, USA) was added followed by incubation at 37 °C for 1 hour. Chromogenesis was performed as described above.

### Western blot

Commercial IgA1 or purified IgA1 from IgAN or non-IgAN patient serum or artificial IgA1-IgG immune complexs was resolved in 12% SDS-PAGE gel and transferred to PVDF membrane (Minipore, USA). After 1-hour’s blocking with 2.5% bovine serum albumin, HRP-conjugated mouse anti-human IgA1 monoclonal antibody (Fc fragment specific, 1:1000, Abcam, USA) or HRP-conjugated donkey anti-goat IgG (1:1000, Santa Cruze, USA) was employed to recognize the total IgA1 at 4 °C overnight. The membrane was then washed with TBST to remove unbound antibody followed by signal development with Immobilon Western Chemiluminescent HRP Substrate (Minipore, USA). For detection of galactose-deficient IgA1 by western blot, the blot was firstly treated with 15 mU/ml neuraminidase for 6 hours at 37 °C after membrane transfer. After washing with TBST, biotinylated HAA (1:200, Sigma, USA) was used to bind the GalNAc-exposed IgA1 overnight at 4 °C and HRP-conjugated avidin (1:5000, Sigma, USA) was introduced to recognize biotin for 1 hours at 37 °C. Signal development was the same as above. Gray intensity of western blot signal band was calculated with Image J 1.47V software (NIH, USA).

### Immunofluorescence and Fluorescent HAA Staining

Kidney biopsies from patients and specimens from mice were sectioned at 4 μm with a Leica cryostat. After rehydration, sections were fixed in 4% paraformaldehyde (PBS) for 10 min and blocked in 3% BSA (in PBS) for 1 hour. Then, the sections were incubated with fluorescence-labeled antibody at indicated dilution ratio at 37 °C for 2 hours or 4 °C overnight. Unbound antibodies were removed by PBS washing and sections were mounted with 80% glycerol. Antibody used are the following, FITC-conjugated mouse monoclonal anti-human IgA1 Fc (1:50, Abcam, UK), FITC-conjugated AffiniPure Rabbit Anti-Human IgA, F(ab’)2 Fragment Specific (Jackson ImmunoResearch, USA), Rhodamine (TRITC)-conjugated AffiniPure Rabbit Anti-Goat IgG, F(ab’)2 Fragment Specific (Jackson ImmunoResearch, USA). For fluorescent HAA staining, the sections were pre-treated with 15 mU/ml neuraminidase at 37 °C for 2 hours after blocking with 3% BSA. Thereafter, Texas Red-HAA (1:50, USBiological, USA) binding was performed at 37 °C for 2 hours. After rinsing to remove unbound HAA, the sections were mounted as above. Photography was taken with Nikon ECLIPSE50i microscope (Japan) and further processed with Adobe Photoshop CS5 software. The fluorescent intensity was quantified with Image J 1.47V software (NIH, USA).

### *In vitro* and *In vivo* Experiment with Passive IgAN Mouse Model

BALB/c mice (aged 8–10 weeks, 22–25 g, male) were injected with 700 μg of IgA1-IgG or agIgA1(agIgA1)-IgG immune complexes (in 1 ml of sterile PBS) via tail vein. For control, only saline or IgA1 or a mixture of human IgA1 and non-specific goat IgG was delivered at equivalent amount and volume. Mice were anaesthetized with pentobarbital sodium and sacrificed at indicated time. Kidney was harvested, snap-freezed in liquid nitrogen and sectioned at 5 μm followed by quantification of deposition of immune complexs by immunofluorescence. For *in vitro* IgA protease digestion assay, section were firstly digested with indicated IgA protease solution (0.01 mg/ml IgA protease in 50 mM Tris-HCl, 100 mM NaCl and 1 mM EDTA. pH 7.5) after rehydration. Then, section was rinsed with PBS and subjected to standard immunofluorescence assay. For *in vivo* IgA protease treatment, the established passive IgAN mice were intravenously administrated with indicated amount of purified bacterial IgA protease (in 1 ml of sterile PBS) at 6 hours post immune complexs injection. 2 hours post IgA protease delivery, mice were sacrificed and kidney was sampled as above. The amount of deposited immune complexes was determined by immunofluorescence as previously described. All animal experiments were approved and conducted in accordance to the guidelines issued by Ethics Committee for animal experimentation of Southwest Medical University.

### Statistical analysis

All quantitative data were checked for their normal distribution and presented as mean ± SEM and were analyzed by Two-way ANOVA analysis followed by multi-group comparisons using SPSS 13.0 software. Statistic graphs were generated with GraphPad Prism 5 software. P value < 0.05 was considered to be statistically significant.

## Additional Information

**How to cite this article**: Wang, L. *et al*. Bacterial IgA protease-mediated degradation of agIgA1 and agIgA1 immune complexes as a potential therapy for IgA Nephropathy. *Sci. Rep.* **6**, 30964; doi: 10.1038/srep30964 (2016).

## Supplementary Material

Supplementary Information
