# Supplementary material for: Bacterial IgA protease-mediated degradation of agIgA1 and agIgA1 immune complexes as a potential therapy for IgA Nephropathy
Source: Sci Rep. 2016 Aug 3;6:30964. doi: 10.1038/srep30964 (PMC4971536; doi:10.1038/srep30964)
Supplement: Supplementary Information [file srep30964-s1.pdf]

Supplementary information

Bacterial IgA protease-mediated degradation of agIgA1 and agIgA1 immune complexes as a potential therapy for IgA Nephropathy

Li Wang[1,2,+], Xueying Li[3,+], Hongchun Shen[4], Nan Mao[5], Honglian Wang[1], Luke Cui[6], Yuan Cheng[7], Junming Fan[2,3,8,*]

[1]Laboratory of Organ Fibrosis Prophylaxis and Treatment by Combine Traditional Chinese and Western Medicine, Research Center of Combine Traditional Chinese and Western Medicine, Affiliated Traditional Medicine Hospital of Southwest Medical University, Luzhou, Sichuan, 646000, China.

[2]State Key Laboratory of Biotherapy, West China Hospital, Sichuan University, Chengdu, Sichuan, 610041, China.

[3]Department of Nephrology, The Affiliated Hospital of Southwest Medical University, Luzhou, Sichuan, 646000, China.

[4]College of Integrated Chinese and Western Medicine, Southwest Medical University, Luzhou, Sichuan, 646000, China.

[5]Department of Nephrology, The First Affiliated Hospital of Chengdu Medical College, Chengdu, Sichuan, 610041, China.

[6]Department of Nephrology, The Third People's Hospital of Chengdu, Chengdu, Sichuan, 610041, China.

[7]Department of Nephrology, Shenzhen Second People's Hospital, The First Affiliated Hospital of

Shenzhen University, Shenzhen, 518000, China

[8]Department of Nephrology, The Affiliated Traditional Chinese Medicine Hospital of Southwest Medical University, Luzhou, Sichuan, 646000, China.

[+]These authors contributed equally to this work.

[*]Corresponding author: Junming Fan, State Key Laboratory of Biotherapy, West China Hospital, Sichuan University at 1# Keyuan 4 Road, Gaopeng Street, Chengdu, 610041, P.R. China. Phone: +86-13808285633. Fax:+ 86-028-85125449. E-mail: junmingfan@163.com

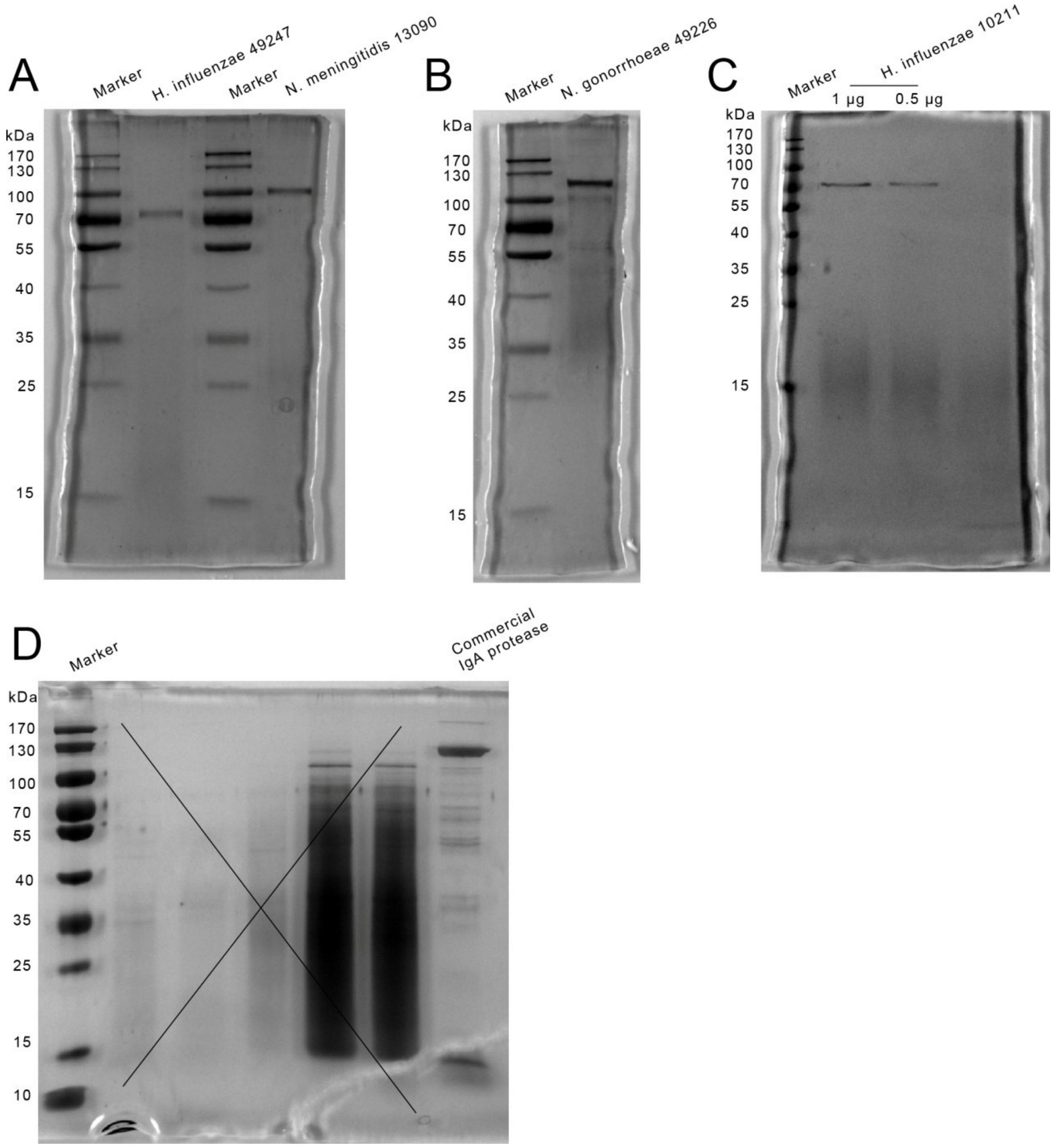

Figure S1. Purity validation of IgA protease purifed from *H. influenzae* 49247, *H. influenzae* 10211, *N. gonorrh*oeae 49247 and N. meningitidis 13090. 1µg of purified IgA protease from *H. influenzae* 49247 (A), *H. influenzae* 10211 (C, both 0.5 and 1µg), *N. gonorrhoeae* 49226 (B), *N. meningitidis* 13090 (A) and commercial IgA protease (D) derived from *N. gonorrhoeae* were resolved, respectively, in SDS-PAGE gel and visualized by silver staining. In D, the irrelative lanes were marked by corss lines.

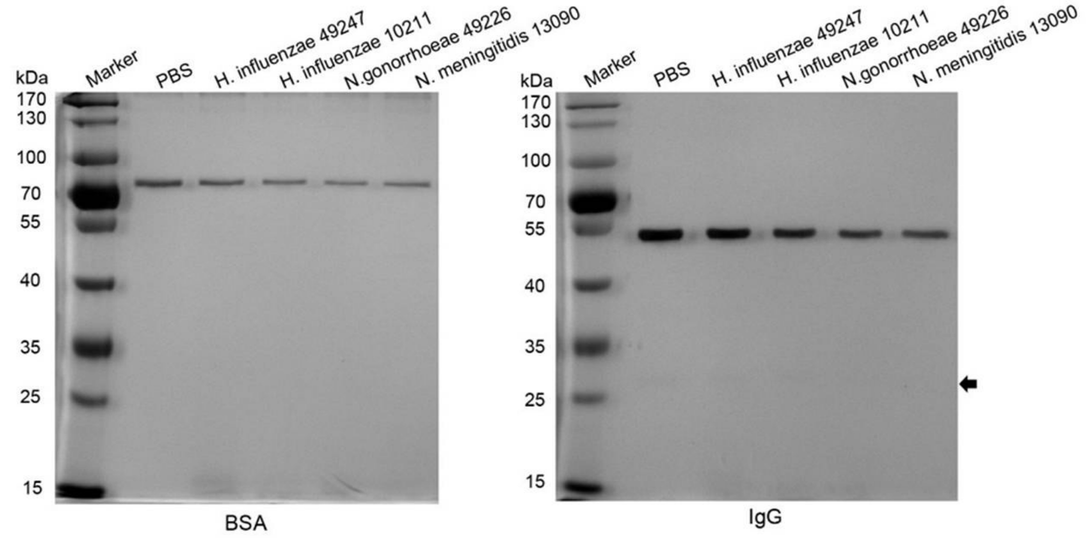

Figure S2. Digestion of BSA and IgG by IgA proteases. 0.5 μg of BAS or mouse IgG was incubated with PBS or 0.05 μg of indicated IgA protease overnight at 37 °C. The digestion mixture was subjected to SDS-PAGE analysis and sliver staining. light chain of IgG which can be slightly observed (arrow).

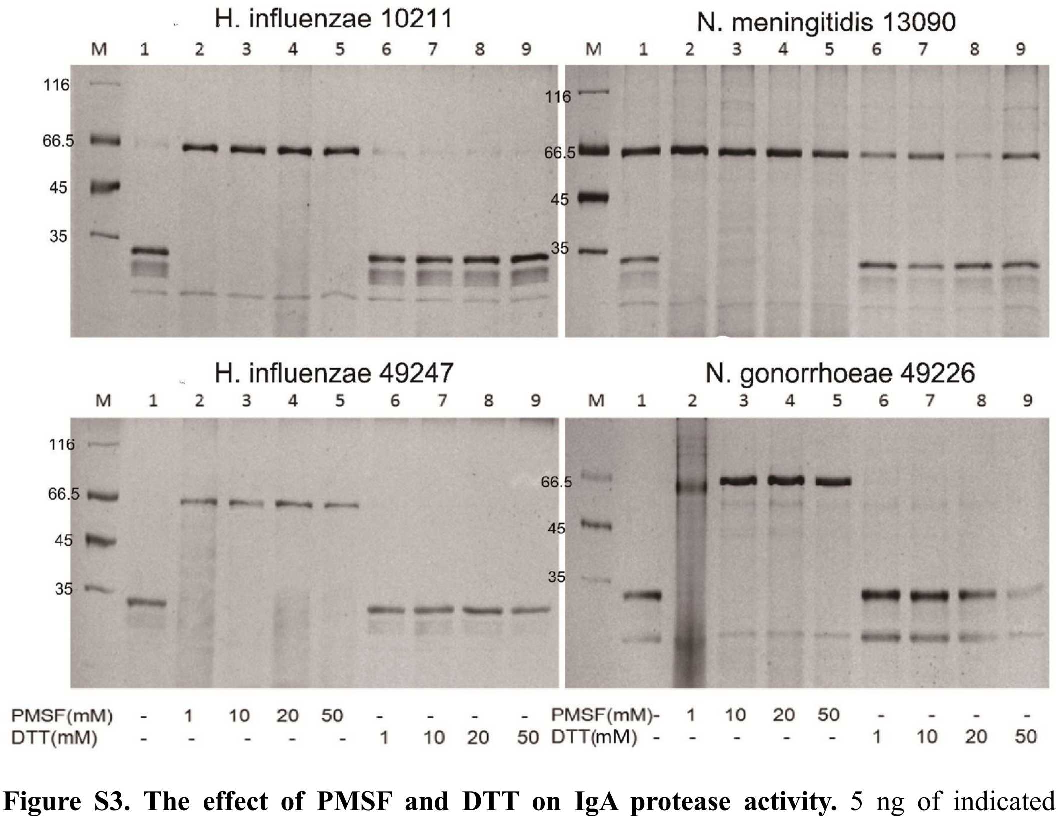

Figure S3. The effect of PMSF and DTT on IgA protease activity. 5 ng of indicated IgA proteases were pre-incubated in the presence of indicated concentration of PMSF and DTT for 1 h at 37 °C followed by addition of 0.5 μg of IgA1 substrate and incubation for additional 2 h at 37 °C. Digestion pattern was revealed with SDS-PAGE gel and silver staining. Note the digestion

pattern in panel of *N. meningitidis* 13090 that addition of DTT (lane 6-9) showed no remarkable influence on the digestion pattern of substrate IgA1 compared with control (lane 1).

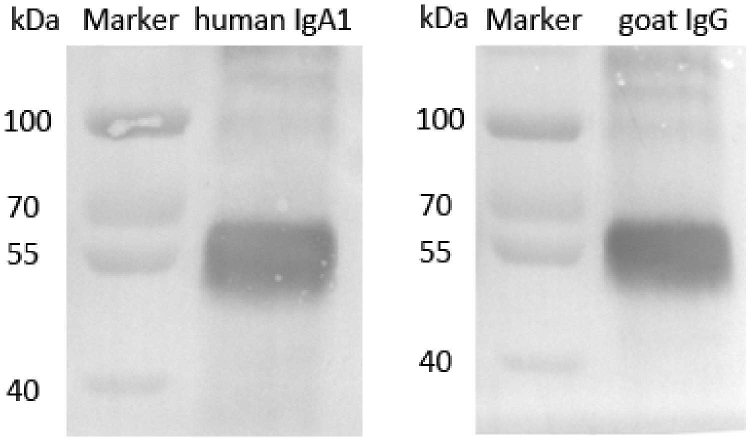

Figure S4. Immunoblotting to detect the IgA1 and IgG components in artificial IgA1-IgG immune complex. 0.5 μg of immune complex was loaded each lane. IgA1 was probed by HRP-conjugated mouse anti-human IgA1 monoclonal antibody (Fc fragment specific) and IgG (Fab) was probed by HRP-conjugated donkey anti-goat IgG antibody. Note that the signal was developed with DAB and only heavy chain was shown for IgA1 and IgG.

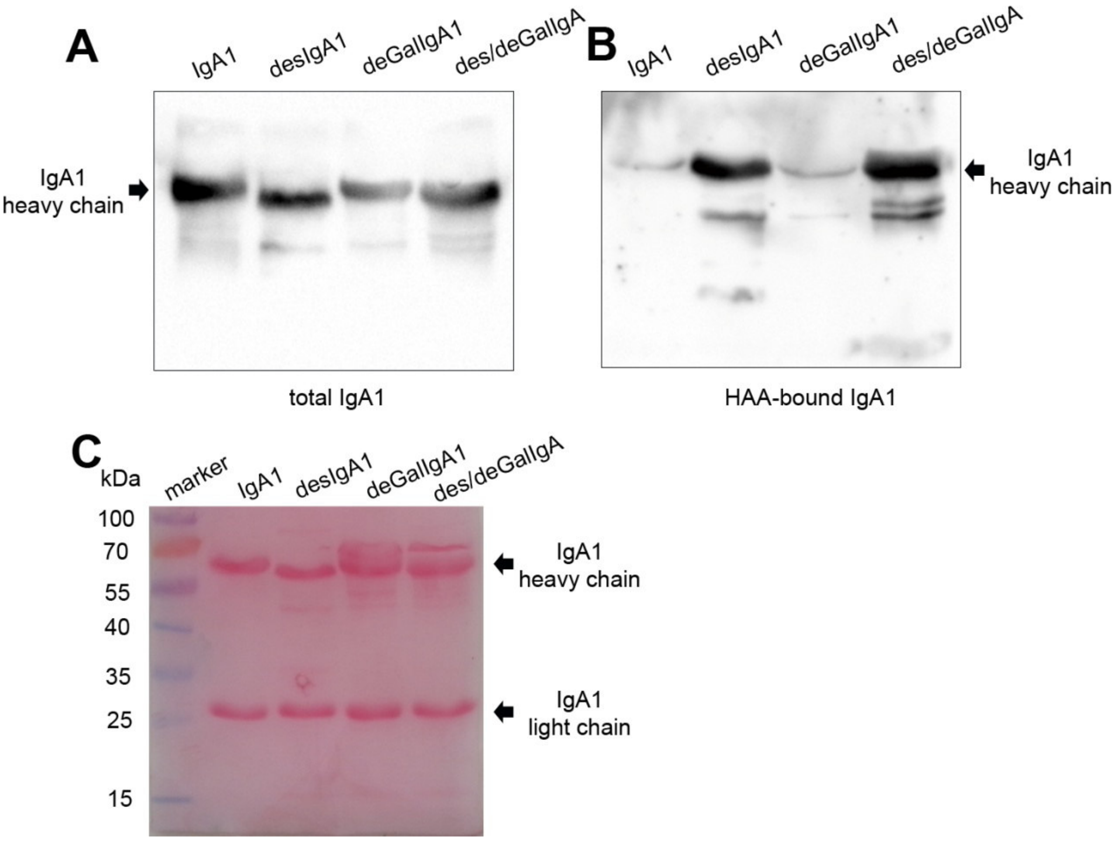

Figure S5. Determination of the glycosylation status of IgA1 treated with deglycosylation

enzyme. 5 µg of human IgA1 treated with different deglycosylation enzyme was subjected to western blot analysis. The heavy chain, revealed by heavy chain-specific antibody, was used to represent the total IgA1 load (**A**). The galactose-deficient IgA1 was recognized by HAA binding (**B**). The blot was further stained with Ponceau S solution to validate the equal loading amount of IgA1 (**C**).

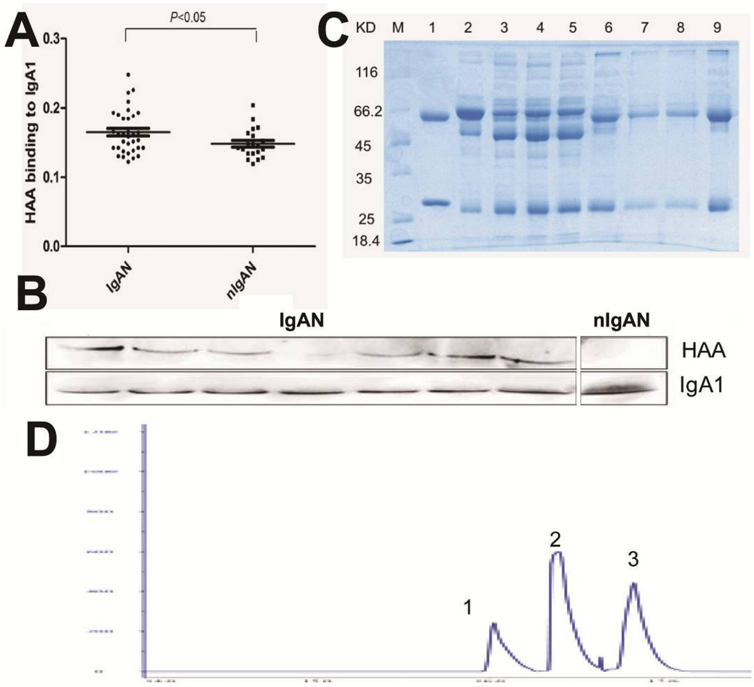

Figure S6. Purification of IgA from serum of IgAN and non-IgAN patients. the serum glycosylation status of IgAN and non-IgAN patients was determined by HAA-based ELISA (**A**). The HAA binding intensity was normalized by the level of total IgA1. **B**. HAA-binding based western blot was used to determine the glycosylation status of sera from IgAN and non-IgAN patient. Sera from IgAN patients showing high level of aberrant glycosylation of IgA1 were pooled for IgA1 purification (**C**). lane 1 is commercial human IgA1 used as control, lane 2 diluted serum, lane 3 raw protein from $(NH_4)_2SO_4$ precipitation, lane 4 pass-through serum after Jacalin column absorption, lane 5 wash buffer, lane 6-8 successive elution with 0.1 M melibiose in PBS, lane 9 pooled and concentrated elute. **D**. Size-restricted chromatography with Sephadex G-200 column to separate IgA1 of different isoforms. 1 is polymeric IgA1, 2 monomeric IgA1 and 3 non-IgA protein or impurity.

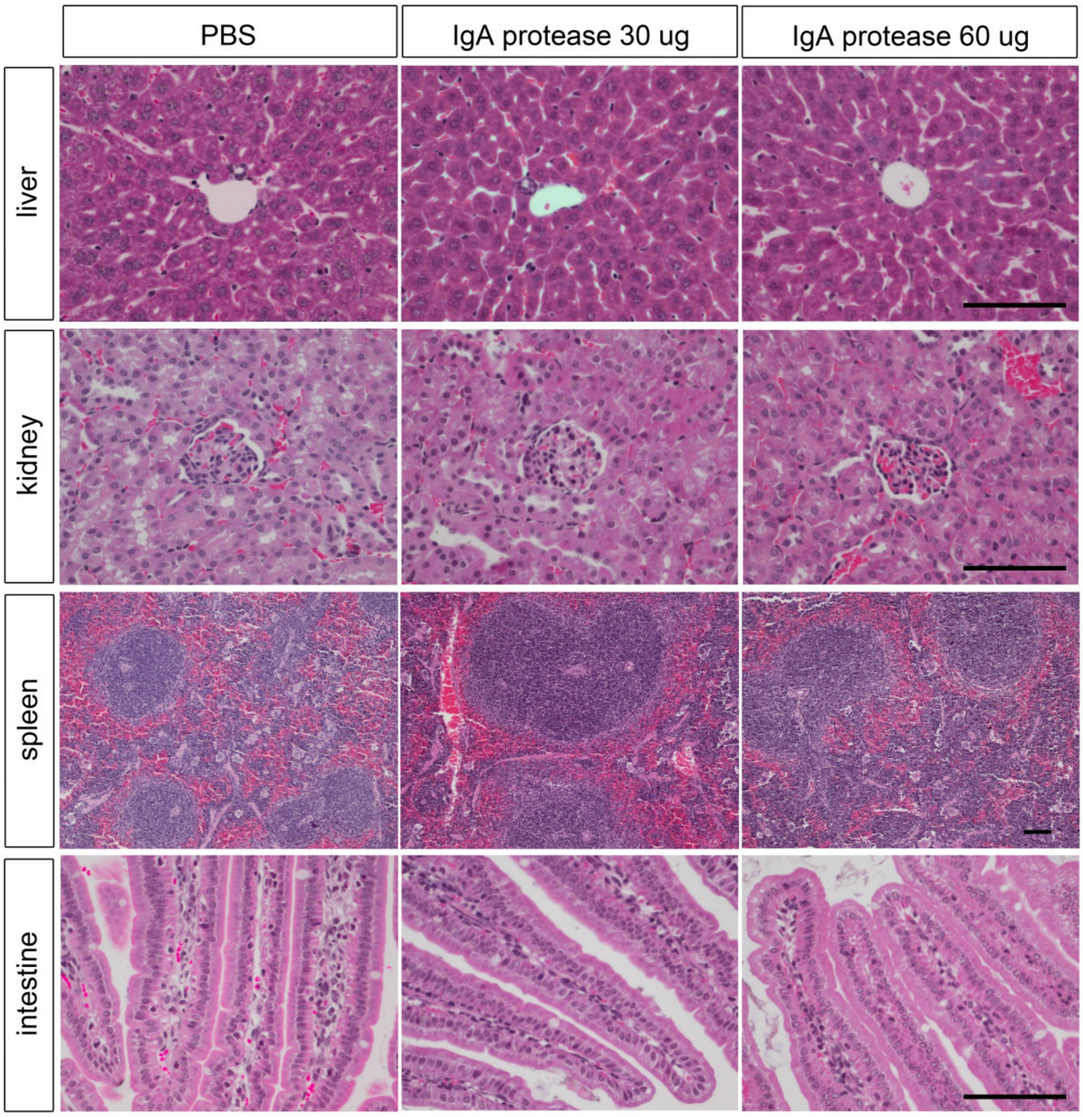

Figure S7. Histopathology analysis (HE staining) of liver, kidney, spleen, intestine from BALB/c mice 24 hours post injection of PBS or *H. influenzae* 49247 IgA protease (30 μg or 60 μg each mouse). Scale bar, 100um.

Table S1. Toxicity effect of IgA protease in respect to serological items in BALB/c mice

	PBS (n=4)	30 μg IgA protease/mouse (n=4)	60 μg IgA protease/mouse (n=5)
TP, g/L	44±2.94	46.25±2.98^{ns}	46.6±1.34^{ns}
ALB, g/L	24±2.16	24.5±2.08^{ns}	24.8±0.84^{ns}
AST, U/L	109.5±20.33	118.75±8.3^{ns}	117±26.5^{ns}
ALT, U/L	31.5±1.29	42±9.2^{ns}	38.2±3.90^{ns}
TG, mmol/L	1.13±0.18	0.81±0.22^{ns}	0.91±0.19^{ns}
BUN, mmol/L	6.93±0.85	7.98±0.78^{ns}	8.28±0.86^{ns}
CREA, umol/L	12.75±4.11	15.5±3.1^{ns}	15.8±3.03^{ns}

Glu, mmol/L	7.34±1.53	6.21±2.37 ns	6.99±2.2 ns
Cys-c, mg/L	0.24±0.02	0.24±0.01 ns	0.24±0.01 ns
eGFR, ml/min	315.95±26	318.32±16.89 ns	314.07±10.54 ns
AMS, U//L	695.25±49.18	776.25±72.2 ns	790.6±97.7 ns

Abbreviations: TP, total protein; ALB, serum albumin; AST, glutamic-oxaloacetic transaminase; ALT, glutamic-pyruvic transaminase; TG, triglyceride; BUN, blood urea nitrogen; CREA, creatinine; Glu, glucose; Cys-c, cystatin C; eGFR, estimated glomerular filtration rate; AMS, amylase. ns, no statistically significant difference vs PBS group.

Table S2. Culture condition for each bacterial species used in this study

bacteria	Solid culture plate	Liquid culture medium	Culture condition
N. gonorrhoeae	Columbia chocolate agar plate	Brain-heart infusion broth	37 °C, 5% CO2, 200rpm
H. influenzae	Columbia chocolate agar plate	Brain-heart infusion broth supplemented with 10 μg/ml Hemin and 10ug/ml β-NAD	37 °C, air condition, 200rpm
N. meningitidis	Columbia chocolate agar plate	Brain-heart infusion broth	37 °C, air condition, 200rpm
S. pneumoniae	Columbia chocolate agar plate	Brain-heart infusion broth	37 °C, air condition, 200rpm
S. mutans	Brain-heart infusion agar plate	Brain-heart infusion broth	37 °C, anaerobic (80% N_2, 10% CO_2, 10%H_2), 200rpm
E. coli	LB agar plate	LB broth	37 °C, air condition, 200rpm
